# Foliar application of chitosan-putrescine nanoparticles (CTS-Put NPs) alleviates cadmium toxicity in grapevine (*Vitis vinifera* L.) cv. Sultana: modulation of antioxidant and photosynthetic status

**DOI:** 10.1186/s12870-023-04420-7

**Published:** 2023-09-04

**Authors:** Sima Panahirad, Gholamreza Gohari, Gholamreza Mahdavinia, Hessam Jafari, Muhittin Kulak, Vasileios Fotopoulos, Rubén Alcázar, Mohammadreza Dadpour

**Affiliations:** 1 Department of Horticultural Sciences, Faculty of Agriculture, University of Tabriz, Tabriz, Iran; 2 Department of Horticultural Sciences, Faculty of Agriculture, University of Maragheh, Maragheh, Iran; 3 Polymer Research Laboratory, Department of Chemistry, Faculty of Science, University of Maragheh, Maragheh, Iran; 4 Department of Herbal and Animal Production, Vocational School of Technical Sciences, Igdir University, Igdir, Turkey; 5 Department of Agricultural Sciences, Biotechnology and Food Science, Cyprus University of Technology, Limassol, Cyprus; 6 Department of Biology, Healthcare and Environment, Faculty of Pharmacy and Food Sciences, University of Barcelona, Barcelona, Spain

**Keywords:** Heavy metal, Toxicity, Nanoparticles, Carrier, Abiotic stress, Horticultural crop

## Abstract

**Background:**

Cadmium (Cd) stress displays critical damage to the plant growth and health. Uptake and accumulation of Cd in plant tissues cause detrimental effects on crop productivity and ultimately impose threats to human beings. For this reason, a quite number of attempts have been made to buffer the adverse effects or to reduce the uptake of Cd. Of those strategies, the application of functionalized nanoparticles has lately attracted increasing attention. Former reports clearly noted that putrescine (Put) displayed promising effects on alleviating different stress conditions like Cd and similarly chitosan (CTS), as well as its nano form, demonstrated parallel properties in this regard besides acting as a carrier for many loads with different applications in the agriculture industry. Herein, we, for the first time, assayed the potential effects of nano-conjugate form of Put and CTS (CTS-Put NP) on grapevine (*Vitis vinifera* L.) cv. Sultana suffering from Cd stress. We hypothesized that their nano conjugate combination (CTS-Put NPs) could potentially enhance Put proficiency, above all at lower doses under stress conditions via CTS as a carrier for Put. In this regard, Put (50 mg L^− 1^), CTS (0.5%), Put 50 mg L^− 1^ + CTS 0.5%” and CTS-Put NPs (0.1 and 0.5%) were applied on grapevines under Cd-stress conditions (0 and 10 mg kg^− 1^). The interactive effects of CTS-Put NP were investigated through a series of physiological and biochemical assays.

**Results:**

The findings of present study clearly revealed that CTS-Put NPs as optimal treatments alleviated adverse effects of Cd-stress condition by enhancing chlorophyll (chl) *a*, *b*, carotenoids, ^*Fv*^*/*_*Fm*_, Y(II), proline, total phenolic compounds, anthocyanins, antioxidant enzymatic activities and decreasing Y (NO), leaf and root Cd content, EL, MDA and H_2_O_2_.

**Conclusions:**

In conclusion, CTS-Put NPs could be applied as a stress protection treatment on plants under diverse heavy metal toxicity conditions to promote plant health, potentially highlighting new avenues for sustainable crop production in the agricultural sector under the threat of climate change.

## Background

Contamination of ecosystems with the heavy metals is of critical concerns and the accumulation of the metals in crops and ultimately humans is strictly under investigation [1–3]. According to search on Web of Science using term “cadmium stress”, 3.721 documents in “plant science” category was recorded and 32.36% of those documents were disseminated in last five years (2018–2022) [4]. Authors underline the increasing concerns on contamination and potential risk of cadmium (Cd) on ecosystem components. Heavy metals impose two main issues to be considered. The first one is about its adverse effects on plant growth and health and finally productivity. The second concern is about its accumulation in crops and its fate in other components of the ecosystem. In this regard, any attempts to alleviate the uptake and translocation of the heavy metals through the plant organs are substantially critical. As a abiotic stress factor, Cd is a mobile and water-soluble heavy metal frequently in Cd^2+^ form [5–7], being reported to have no crucial biological roles [7, 8] but having toxic effects on plants and humans [6, 9].

A plethora of documents has clearly reported the adverse effects of Cd on biochemical and metabolic processes of plants. The impacts of Cd stress are translated into critical modifications in agronomic traits, photosynthesis pigments and parameters, membrane integrity, water balance, essential nutrients uptake, proline, total phenolic compounds and antioxidant enzymes activities [5, 10]. For instance, Cd-mediated disturbances in metal homeostasis are manifested as iron deficiency, which in turn results in impairments in the biosynthesis of chlorophylls (Chl), the formation of Chl–protein complexes and the development of thylakoid membranes [7].

In addition, Cd negatively affects the structure of roots and organelles [9, 10]. Furthermore, Cd induces reactive oxygen species (ROS) generation, which is then reflected as oxidative stress. Depending on severity of Cd stress, Cd stress might display critical effects on DNA, proteins and causes membrane damage. Perturbed alterations in cellular responses of the plants are manifested as reduced plant growth and productivity [5, 8, 10].

In order to make the crop plants compatible with stress conditions or to enhance the resilience of the plants, uses of nanoparticles have been great interest in agriculture [11]. With respect to the alleviation of potential damage of Cd in crop plants, the researchers have attempted to investigate the effects of various particles [12–14]. In particular, since Cd toxicity is becoming progressively prevalent, posing a global environmental hazard, various approaches have been applied to reduce its adverse effects on plants with great achievements regarding the use of functionalized nanoparticles and chitosan (CTS) as carrier. For instance, CTS-Se NPs were successfully applied to mitigate Cd toxicity effects [5]. For that reason, our further researches were addressed on potential uses nanoparticles using CTS as a carrier to mitigate Cd toxicity.

Chitosan (CTS) increases chlorophyll content and nutrient uptake of plants and has beneficial effects on plant growth and development [5]. CTS has elicitor-like activity, particularly in the content of phenolic compounds including anthocyanins, and in improving the antioxidant capacity via enhanced activity of phenylalanine ammonia-lyase (PAL) [15, 16]. In addition, CTS has heavy metal ion chelation ability, a remarkable property to deal with heavy metal toxicity [5, 17]. CTS has attracted attention owing to its promising uses in numerous delivery systems in agriculture, particularly as carrier and adsorption matrix for growth-promoting and stress-protection compounds [5, 18, 19], owing to distinct chemical and biological characteristics (e.g., polycationicity, biocompatibility and biodegradability). Importantly, CTS is a non-toxic compound for humans. Carriers like CTS cause slow and prolonged release of protective compounds thus enhancing effectiveness [18]. CTS is also capable of loads protection from undesired environmental situations and loads insistent release from its matrix and prevention of the destructive influences of loads burst release on the plant’s cells. Furthermore, CTS nanoparticles (NPs) have been used for the systematic release of loads through improved beneficial characters in the nano-form leading to improved proficiency and controlled transfer of any loads previously confirmed specifically in selenium [5, 19], and phenylalanine [15].

Putrescine (Put), a major polyamine (PA), has polycationic property [20, 21] with prominent impacts in preserving membranes and negatively charged macromolecules of cells [22, 23]. Put plays vital roles in growth, differentiation and several biological and developmental processes (e.g., cell division, DNA and protein synthesis) of plants [22, 24]. Importantly, Put plays vital roles in plant responses to stress conditions and its concentration increases under stress [6, 21, 22, 24]. The protective effect of Put is presumed to be caused by its effect on protein homeostasis, osmolyte accumulation, stabilization of cellular structures, neutralizing free radicals, modulation of ion channels, activation of the antioxidative machinery, molecular chaperone activity, maintenance of the cation-anion stability, energizing cells by enhancing ATP synthesis and upregulation of stress-related genes [20, 21]. In fact, Put treatment enhances plant performance under stress conditions through improving photosynthetic parameters and maintaining chlorophyll content [23]. Interestingly, Put has essential functions in DNA-protein and protein-protein interactions [6]. Overexpression of PA biosynthetic genes results in increased protection against stresses with a connection to the level of PA increase [21]. Indeed, Put is capable to buffer the decrease in growth, preserves cell membrane integrity, decreases lipid peroxidation, ROS generation and accumulation and chlorophyll loss caused by stress conditions, through enhancement in the expression of genes involved in osmotic adjustment, activity of some antioxidant enzymes and compounds and compatible osmolytes [23]. PAs interact with different metabolic routes and hormone pathways, subsequently resulting in stress tolerance [24]. Put has critical roles in combating heavy metal toxicity and acts as a metal chelator [25, 26]. Put application mitigated the adverse effects of Cd on plants. Likewise, Put concentration increased under Cd toxicity due to higher activity of some enzymes involved in its biosynthesis [25, 27]. PAs are able to reverse oxidative stress that is induced by heavy metals, mainly through the activation of the antioxidant machinery [26, 28, 29]. Carbon quantum dots (CQDs) were effectively applied as carrier for Put to improve its efficiency at alleviating salinity stress [23]. Thus, Put could be used as potential load for other carries like CTS with further improved impacts.

In the categories of horticultural crops, grapevine (*Vitis vinifera* L.) is of the oldest fruit crops and native to the only Mediterranean/Western Asiatic regions [30], being characterized with two productive organs (fruit berry and seeds) [23]. The crop has an array of significant uses due to the flavonoids as representative of soluble phenolics [31]. As of the most crop and non-crop species, grapevine also suffers from abiotic and biotic stress factors [31–35], in general and Cd stress [4, 36]. As noted in our very recent study [4], novel approaches regarding buffering the adverse effects of Cd stress and subsequently ensuring the plants to reach to the fruit and seed onset are needed. For example, Ramzan et al. [37] demonstrated that the combined use of zinc oxide nanoparticles (Zn NPs) and *Moringa oleifera* leaf extract effectively mitigated Cd toxicity in *Linum usitatissimum*. This was achieved by enhancing the activities of antioxidant enzymes and promoting osmolyte accumulation in the leaves. The synergistic application of *M. oleifera* leaf extract and ZnO NP protected photosynthesis and ameliorated the negative effects Cd toxicity, thereby reducing oxidative stress. In this context, we addressed the present study on application of newly synthesized nanoparticles in grapevine plants suffering from Cd stress.

Considering the affirmative functions of Put and CTS on cellular responses of plants and efficiency of CTS as a carrier, we hypothesized that the conjugated nano-form (CTS-Put NPs) could likely further increase their effects in a synergistic tenet and also could improve Put effectiveness via better entrance in plant cells. Corresponding to significant alterations in cellular responses of grapevine plants after applications of CTS-Put NPs, it was predicted that adverse effects of Cd stress on grapevine plant could be buffered and consequently crop productivity could be ensured. Therefore, the study was directed to a theoretical framework in relation to the NPs treatment, specifically at lower doses, that would mitigate Cd toxicity effects through improved physiological and biochemical traits of grapevines via enhanced Put effects over nano-delivery by CTS.

## Results and discussion

### Synthesis and characterization of chitosan-putrescine nanoparticles (CTS-Put NPs)

**Fig. 1:**
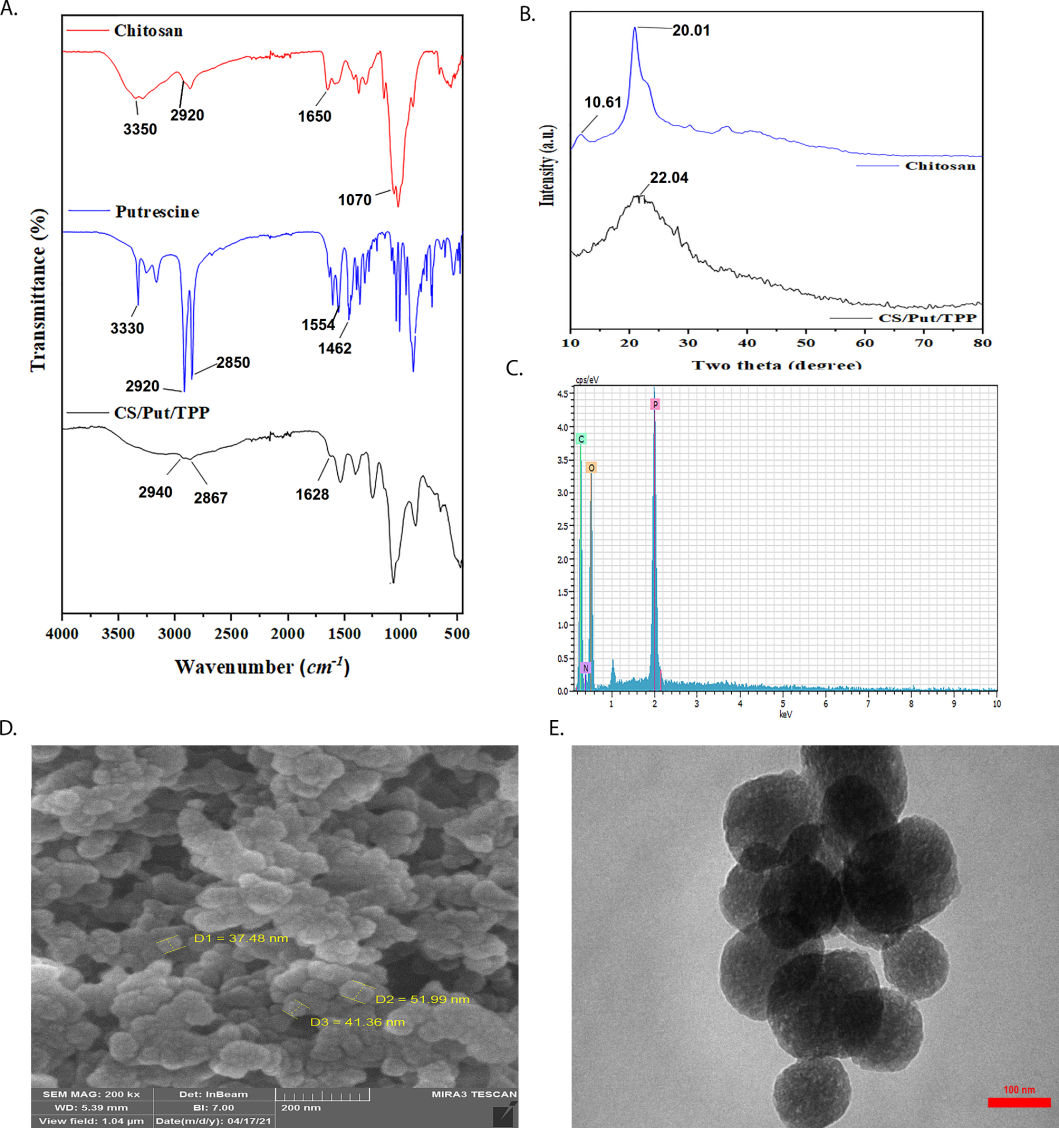
FTIR spectra of chitosan, putrescine, and Put-loaded chitosan nanoparticles (**A**); XRD patterns of chitosan and Put-loaded chitosan nanoparticles (**B**); Energy dispersive X-ray spectroscopy (EDS) (**C**); SEM micrograph of Put-loaded chitosan nanoparticles (**D**); TEM image of Put-loaded chitosan nanoparticles; and EDS spectra of Put-loaded chitosan nanoparticles (**E**)

Structural characterization of CTS, Put, and Put-loaded CTS NPs was investigated by FTIR spectra (Fig. 1A) based on the functional groups and their interactions. In the CTS spectrum, the characteristic bands appeared at 3350, 2920, and 1650 cm^− 1^ which corresponded to the –OH stretching, -C-H stretching and the amide groups on CTS, respectively [38]. In the spectrum of pure Put, the N-H stretching and bending vibrations of amine groups on Put were confirmed by a band at around 3330 and 1570 cm^− 1^, respectively. The bands at 2920 and 2850 cm^− 1^ were assigned to the asymmetrical and symmetrical stretching vibrations of CH_2_ groups on pure Put. In the FTIR spectrum of Put-loaded CTS NPs, some characteristic peaks appeared. Due to the similarity of functional groups of pure CTS and Put, the distinction of their functional groups was not possible, originating from overlapping the CTS and Put characteristic bands. The characteristic peaks of Put-loaded CTS NPs were confirmed with a slight shift compared to raw CTS, which can confirm the successful preparation of Put-loaded CTS NPs. The crystalline structure of pure CTS and Put-loaded CTS NPs were investigated and the results were shown in Fig. 1B. Compared with the XRD of pure CTS which displayed the characteristic peaks at 2θ = 10.61° and 20.01° which belongs to the partial crystalline structure of CTS [39, 40], the Put-loaded CTS NPs indicated a broad peak with low intensity at 2θ = 22.04°. The broad peaks can be related to the CTS-TPP and CTS-Put interactions, resulting in from amorphous structure. In the spectrum obtained from EDS analysis (Fig. 1C), the observed peaks were related to C, N, and O which demonstrated the presence of CTS in the nanocarrier structure. The morphology of Put-loaded CTS NPs was studied by scanning electron microscopy and transmittance electron microscopy. As shown in Fig. 1D, the SEM image showed sphere-like structure. The TEM image displayed clearly the formation of spherical CTS NPs with an average diameter size of 100 nm (Fig. 1E). It may be noted that the diameter size of NPs obtained from SEM image is less than TEM result. This can be attributed to the drying of NPs to record the SEM image.

### Photosynthetic pigments (chl *a*, *b* and carotenoids) and chlorophyll fluorescence parameters (^Fv^/_Fm_, Y (NO) and Y (II))

**Fig. 2:**
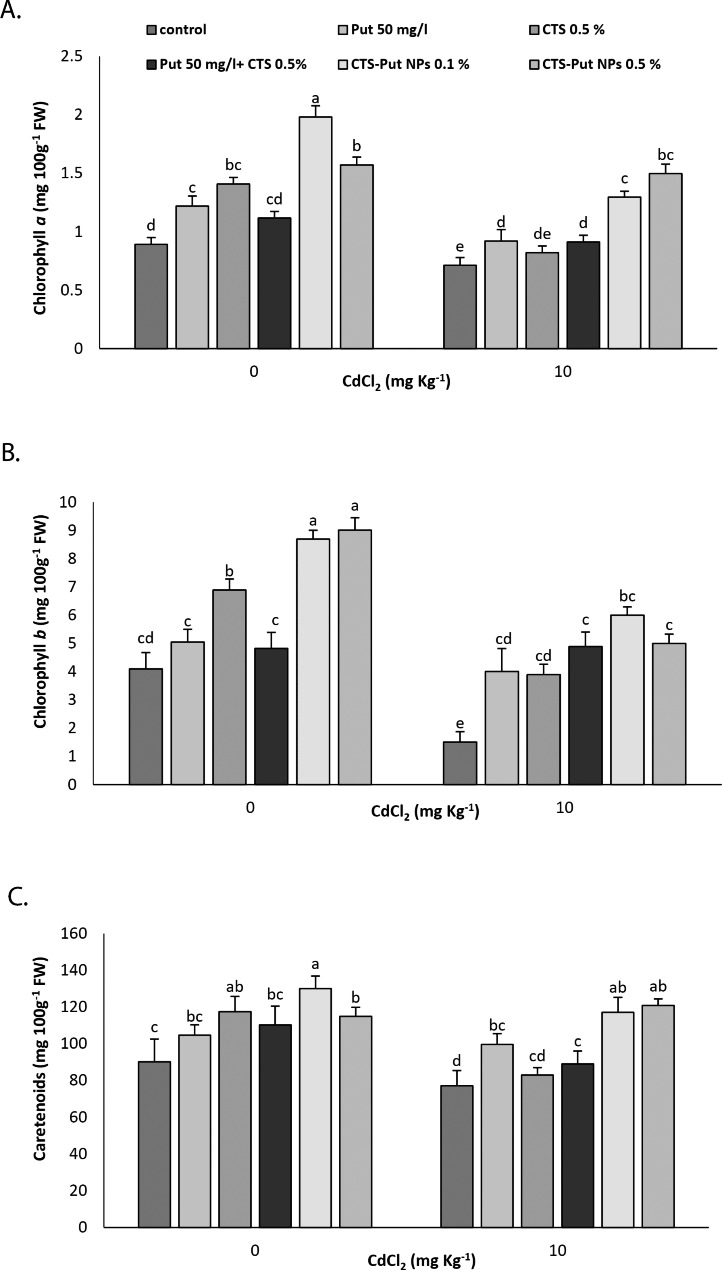
Effect of putrescine (Put; 50 mg L-1), chitosan (CTS; 0.5%), “Put 50 mg L^− 1^ + CTS 0.5%” and “chitosan-putrescine nanoparticles” (CTS-Put NPs; 0.1 and 0.5%) treatments on chlorophyll (chl) *a* (**A**), chl *b* (**B**) and carotenoids (**C**) contents of grape-vines (*Vitis vinifera* L. cv. Sultana) under cadmium (Cd)-stress conditions (0 and 10 mg kg^− 1^). Same letters are not significantly different at *p* < 0.05

**Fig. 3:**
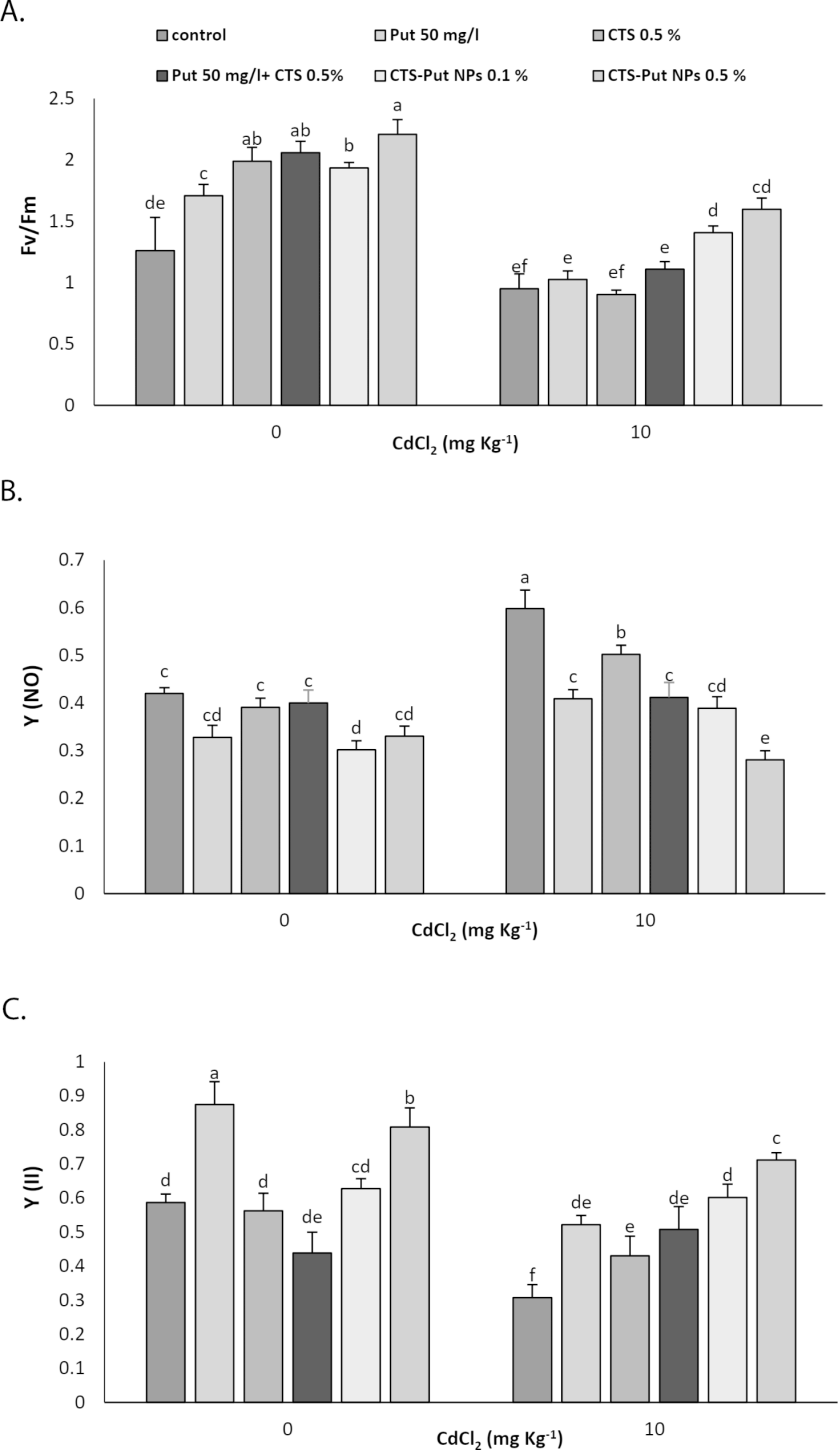
Effect of putrescine (Put; 50 mg L^− 1^), chitosan (CTS; 0.5%), “Put 50 mg L^− 1^ + CTS 0.5%” and “chiosan-putrescine nanoparticles” (CTS-Put NPs; 0.1 and 0.5%) treatments on chlorophyll fluorescence parameters including F_v_/F_m_ (**A**), Y (NO) (**B**) and Y (II) (**C**) of grapevines (*Vitis vinifera* L. cv. Sultana) under cadmium (Cd)-stress conditions (0 and 10 mg kg^− 1^). Same letters are not significantly different at *p* < 0.05

Cd stress adversely affected photosynthetic pigments and fluorescence parameters (*p* < 0.05; Figs. 2 and 3). Of the treatments, all treatments, except Put 50 mg L^− 1^ + CTS 0.5%, increased content of Chl *a* in non-stressed plants. In non-stressed plants, the highest content of Chl *a* was achieved with CTS-Put NPs (0.1%). On the other hand, all treatments, except CTS 0.5%, increased content of Chl *a* in plants suffering from Cd-stress. In Cd-stress submitted plants, the highest content of Chl *a* was recorded at CTS-Put NPs (0.5%). Both (0.1% and 0.5%) CTS-Put NPs treatments increased content of Chl *b* under non-stress conditions whereas all applied treatments had positive effects on content of Chl *b* in plants suffering from Cd stress (Fig. 2B).

Concerning content of carotenoids, either CTS or CTS-Put NPs treatments increased the content under non-stress conditions. On the other hand, all treatments, except CTS, increased the content of carotenoids in plants subjected to Cd-stress. Of the treatments, 0.1% CTS-Put NPs contributed more in content of carotenoid in plants under either stress or non-stress conditions (Fig. 2C).

All treatments significantly increased ^*Fv*^*/*_*Fm*_ in plants grown under non-stress conditions and the relevant value peaked with 0.5% CTS-Put NPs. In Cd-stress submitted plants, either 0.1% or 0.5% CTS-Put NPs positively affected the values of ^*Fv*^*/*_*Fm*_ (Fig. 3A). Considering the values of Y (NO), only 0.1% CTS-Put NPs had positive effects under non-stress conditions. On the other hand, all treatments showed significant effects through reducing the values of Y (NO) in plants suffering from Cd-stress and the best results were recorded at 0.5% CTS-Put NPs (Fig. 3B). Put and 0.5% CTS-Put NPs treatments increased Y (II) under non-stress condition and all treatments increased the values of Y (II) in plants subjected to Cd stress. The highest values were recorded at 0.5% CTS-Put NPs (Fig. 3C).

Fluorescence parameters are routinely tested to evaluate both biotic and abiotic stress. The reduction in ^Fv^/_Fm_ values serves as an indicator of the inhibition of the photosynthetic apparatus, specifically caused by photoinhibition in the immediate vicinity of the PSII reaction centers [41, 42]. Cd, as an operative photosynthetic inhibitor, decreases photosynthetic activity of plants via reduction in Chl content and carbon fixation [5, 9, 43], stomatal closure [44, 45] and injury to the light-harvesting complex (PSI and PSII) [43, 46]. In addition, Cd dampens Fe absorption that is required for plant vital procedures like photosynthesis [5, 9]. Toxic levels of Cd critically decrease the content of Chl *a*, *b* and carotenoids by excessive biosynthesis of ROS, as the cases observed in previous reports [5, 47, 48]. Similarly, being exposed to Cd stress significantly reduces the values concerning chlorophyll fluorescence parameters such as ^Fv^/_Fm_, Y (NO) and Y (II) [5]. Moreover, replacement of Mg by Cd under high levels of Cd in media causes perturbations in photosynthesis and energy dissipation as well as excessive accumulation of ROS such as superoxide and hydrogen peroxide [49]. For example, increases in H_2_O_2_ production declines photosynthesis via damages in Calvin cycle [47]. However, the adverse effects of Cd stress might be buffered with the chitosan nanomaterial. For instance, CTS-Se NPs application increased/improved the content of Chl *a*, *b*, carotenoids, ^*Fv*^*/*_*Fm*_, Y (NO) and Y (II) under non- and Cd- stress conditions [5] and chl *a*, *b*, carotenoids under salt stress [19]. As the case observed here, the partial improvements in values of pigments and chlorophyll fluorescence parameters might be attributed to the possible functions of CTS such as Cd chelating [17], improving essential element uptake and increasing chlorophyll synthesis [50] and increasing cytokinin levels, which in turn are reflected into the augmented Chl biosynthesis [51]. As of the prominent regulators [52], plant polyamines and their conjugated forms are considered to contribute to physiological and biochemical responses of plants in the emergence of stress. Polyamines (PAs) regulate stomatal closure via H_2_O_2_ production and interaction with nitric oxide (NO) signaling, Ca^2+^ and ABA [21]. For instance, in the case of salt stress; Put and Put-CQD NPs increased content of Chl *a*, *b*, carotenoids, ^*Fv*^*/*_*Fm*_ and Y (II) [23]. The similar improvements were also observed under Cd stress. PAs could positively affect the membranes of thylakoids, the PSII, light-harvesting complex II, photosynthetic apparatus and protect the pigments by trapping Cd-produced ROS. Put applications significantly improved Chl content in Cd-stress submitted plants through modulating the Chl synthesis, stomatal conductance and photosynthetic area [53, 54]. Put improves photosynthetic proficiency and chlorophyll content by lowering lipid peroxidation and ROS accumulation and enhancing antioxidant system and compatible osmolytes. Hence, Put improves photosynthetic processes during stress via playing roles in their involved proteins [23].

### Leaf and root cd content

**Fig. 4:**
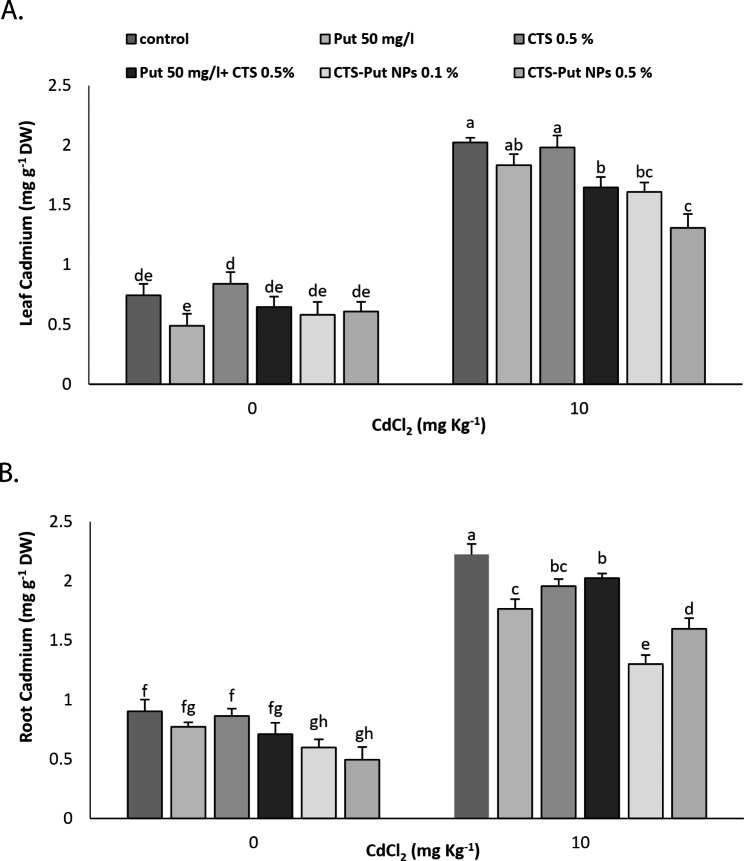
Effect of putrescine (Put; 50 mg L^− 1^), chitosan (CTS; 0.5%), “Put 50 mg L^− 1^ + CTS 0.5%” and “chitosan-putrescine nanoparticles” (CTS-Put NPs; 0.1 and 0.5%) treatments on leaf (**A**) and root (**B**) cadmium (Cd) content of grapevines (*Vitis vinifera* L. cv. Sultana) under cadmium (Cd)-stress conditions (0 and 10 mg kg^− 1^). Same letters are not significantly different at *p* < 0.05

As expected, higher content of Cd was observed in leaf and roots in plant suffering from Cd stress (Fig. 4). In non-stressed plants, the Cd-levels in leaf samples were not significantly affected by the treatments. However, both CTS-Put NPs (0.1% and 0.5%) reduced the lead Cd content in plants subjected to Cd stress (Fig. 4A). Put-CTS NPs treatments reduced the uptake/content of Cd in root in plants grown under non-stress conditions. In Cd-stress submitted plants, all treatments critically reduced the root Cd content and the lowest values of Cd were achieved with 0.1% Put-CTS NPs (Fig. 4B).

Cd is a mobile element and therefore, increases in its content in leaves and root tissues are expected under Cd-stress condition [5, 44, 55]. The increase could be due to Cd entrance via membrane proteins and channels involved in absorption of nutrients [9]. Chitosan based NPs (i.e., CTS-Se NPs) reduced Cd content of leaves and roots under Cd-stress conditions [5]. Similarly, reduction in Cd content was also achieved by Put applications in Cd-stress submitted plants [53]. Such a reduction was explained with the critical roles of Put in combating heavy metal toxicity and acting as a metal chelator [26], which in turn mitigated the adverse impacts of Cd stress on plants [55]. Consequently, Put might reduce Cd entrance via reducing its active form in soil, its transfer in plant and proportion in the cell wall and promote Cd vacuole compartmentalization. Most likely, encouraging impacts of CTS-Put NPs could be described via boosted properties of Put by the NPs very small size and Put persistent release using CTS as a carrier and synergistic effects of Put and CTS in the nano-form.

### MDA and H_2_O_2_

**Fig. 5:**
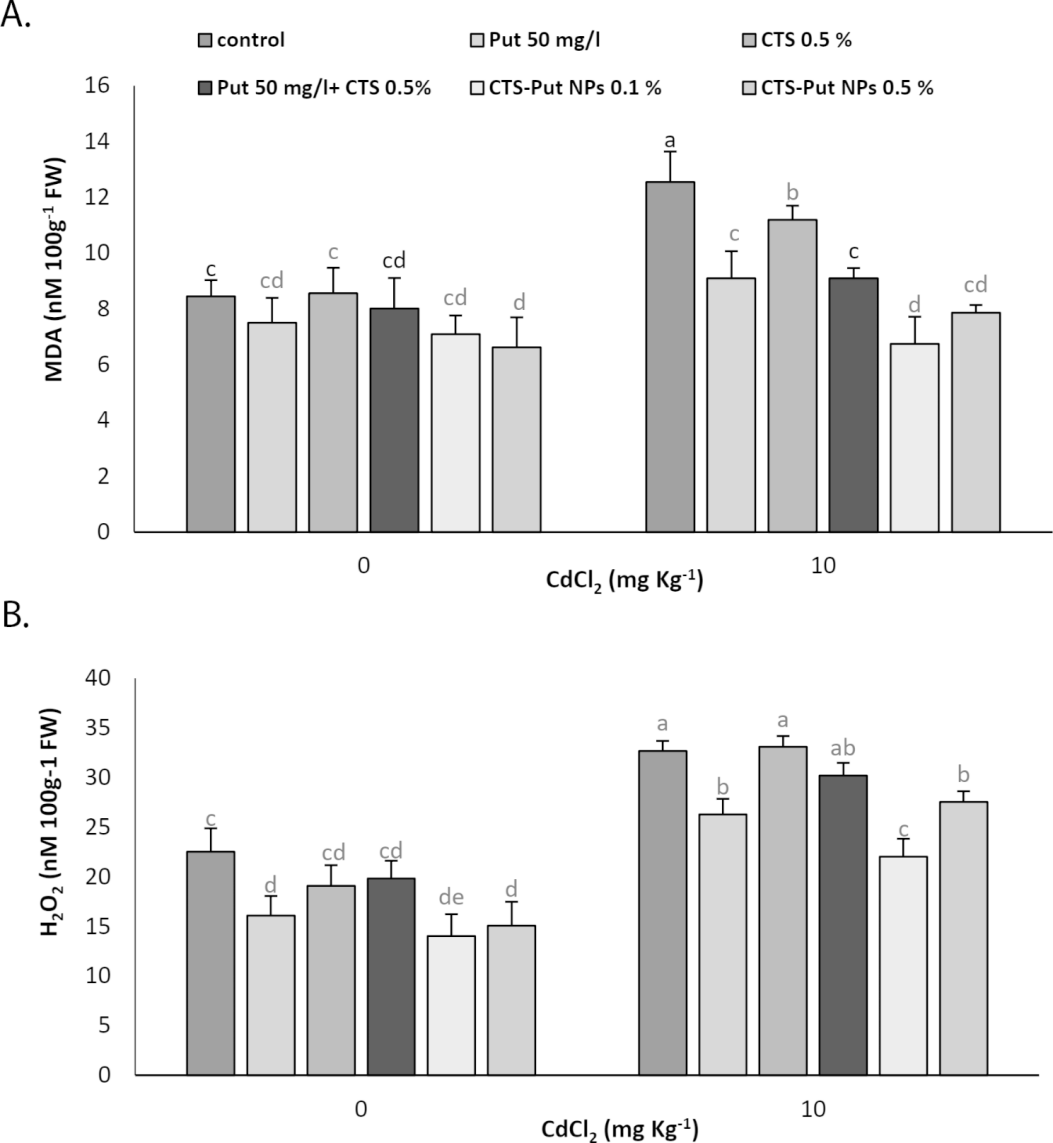
Effect of putrescine (Put; 50 mg L^− 1^), chitosan (CTS; 0.5%), “Put 50 mg L^− 1^ + CTS 0.5%” and “chitosan-putrescine nanoparticles” (CTS-Put NPs; 0.1 and 0.5%) treatments on malondialdehyde (MDA) (**A**) and hydrogen peroxide (H_2_O_2_) (**B**) contents of grapevines (*Vitis vinifera* L. cv. Sultana) under cadmium (Cd)-stress conditions (0 and 10 mg kg^− 1^). Same letters are not significantly different at *p* < 0.05

As expected, the high values of MDA and H_2_O_2_ were observed in Cd-stress submitted plants (Fig. 5). Of the treatments, 0.5% CTS-Put NPs reduced MDA content in plants grown under non-stress conditions. On other hand, all treatments critically decreased MDA content in plants suffering from Cd stress. The values of MDA were achieved with 0.1% CTS-Put NPs (Fig. 5A). Regarding H_2_O_2_ content, Put (50 mg L^− 1^) and CTS-Put NP (0.1 and 0.5%) treatments decreased its values under non- and Cd-stress conditions, with the lowest values at 0.1% CTS-Put NPs (Fig. 5B).

In plant stress studies, MDA is of the crucial indicators for determination of injuries to cell membrane and lipids [48, 55, 56]. Cd stress can increase the content of MDA in plants through the process of lipid peroxidation, which in turn cause cellular damage and disruption [5, 44], as reported for a quite number of crops [5, 44, 48]. In addition to increases in MDA, Cd stress caused increases in H_2_O_2_ content [5, 49, 57]. Cd stress-induced H_2_O_2_ accumulation of might be explained by disorders in electron transference resulting in interrupted photosynthesis and respiration [5, 49]. Very similar to high levels of MDA, H_2_O_2_ induces oxidative stress particularly at higher concentrations, which are in turn manifested as impaired cellular responses of the plants [5]. Likewise, the increases in H_2_O_2_ content and then lipid peroxidation and MDA have been attributed to the interaction between Cd and antioxidant molecules [47, 48]. CTS [5, 47] and CTS-Se NPs [5, 19] applications reduced lipid peroxidation (MDA) in stress-submitted plants probably via possible roles in the activation of antioxidant enzymes. Similar to CTS, Put treatments decreased MDA and H_2_O_2_ content of the plant under Cd-stress conditions and subsequently reduced Cd-induced oxidative stress [58, 59]. Put applications also protected the plants against salt stress through neutralizing free radicals and protecting proteins [60, 61]. In addition, Put applications are considered to contribute to the preservation of cell membranes and macromolecules and structural integrity of cells [21, 22]. Such improvements/protection might be predictors for MDA reduction after Put application in plants suffering from stress. The polycationic nature of Put also makes it capable of maintaining ion balance of cells and preserving DNA, RNA, proteins or membrane lipids by binding to them. For these reasons, PAs (i.e., Put) inhibit lipid peroxidation and ROS production due to their antioxidative roles [21]. As will be discussed below, increases in proline and antioxidant enzymatic activities by Put and particularly CTS-Put NPs treatments could additionally explain the lower H_2_O_2_. High levels of proline could lessen H_2_O_2_ either itself or through triggering the activity of antioxidant enzymes as they could neutralize H_2_O_2_, as well [56, 62]. The lower H_2_O_2_ might be due to lower stress symptoms, independently of the ROS scavenging capacity of Put. Indeed, PAs oxidation also produces ROS, but not all ROS are detrimental, depending on the site of production, levels and signal output. PAs trigger stress protective pathways mediated by ROS [21, 22]. Accordingly, decrease in EL by Put and particularly CTS-Put NPs treatments could be other possible reason for MDA and H_2_O_2_ reduction under Cd-stress condition. CTS-Put NPs could reduce Cd-induced damage to membranes, cellular organelles and biomolecules probably via decreased NADPH-oxidase activity [63, 64].

### EL and proline

**Fig. 6:**
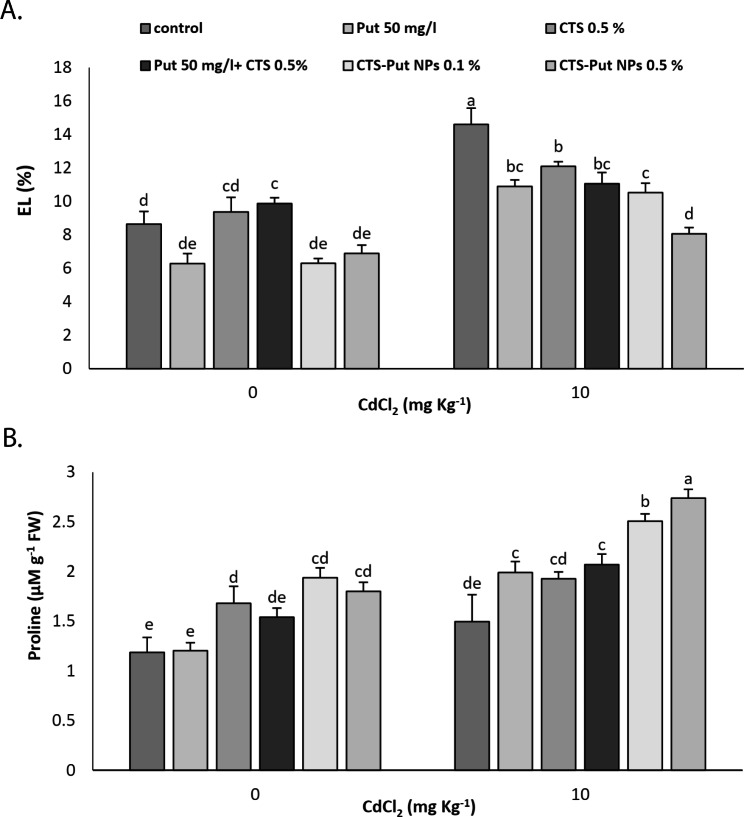
Effect of putrescine (Put; 50 mg L^− 1^), chitosan (CTS; 0.5%), “Put 50 mg L^− 1^ + CTS 0.5%” and “chitosan-putrescine nanoparticles” (CTS-Put NPs; 0.1 and 0.5%) treatments on electrolyte leakage (EL) (**A**) and proline content (**B**) of grapevines (*Vitis vinifera* L. cv. Sultana) under cadmium (Cd)-stress conditions (0 and 10 mg kg^− 1^). Same letters are not significantly different at *p* < 0.05

Cd stress caused increases in EL but did not significantly affect the proline content (Fig. 6). All applications were very effective in decreasing EL in Cd-stress submitted plants and the lowest values were achieved with 0.5% CTS-Put NPs (Fig. 6A). Put and both CTS-Put NPs treatments caused enhanced proline content under non-stress condition. In Cd-stress submitted plants, all applications, except CTS, significantly increased proline content. The values of proline peaked at 0.5% CTS-Put NPs (Fig. 6B).

EL is a universal indicator in stress studies and is linked to the damage to cell membrane stability and integrity [65]. Cd stress firstly damages membrane integrity and subsequently causes increases in EL [5, 22, 66]. However, the adverse effects of Cd stress on EL can be buffered with nanoparticle (i.e. CTS and CTS–Se NPs) [5]. Similarly, Put and Put-CQD NPs decreased EL under non- and salt- stress conditions [23] and prominent functions of Put in stabilizing biological membranes and macromolecular structures of cells were previously confirmed [22, 23]. Put could reduce cell membrane damage, lipid peroxidation and increase osmolytes like proline. Put treatment reduced EL values of the plants subjected to salt stress through direct binding of Put to phospholipid head groups of membranes resulting in stabilizing membranes and preserving their functions [23]. Put positively affected membrane stability of plants under Cd heavy metal toxicity [53, 59]. In addition, Benavides et al. [54] reported that Put application inhibited the negative effect of Cd on membrane integrity. Consequently, affirmative impacts of CTS-Put NPs in this regard could be in line with the above-mentioned reasons and additionally could be related to the enhanced antioxidant enzymes activities that neutralized ROS and lessened membrane damage through superior Put effectiveness in the nano-form.

As an antioxidant osmolyte, proline content boosts under stress conditions. The increases in proline content are, in general, manifested as enhanced tolerance through crucial adjustments in osmotic pressure of cells [5, 15]. Proline preserves protein integrity via acting as a molecular chaperone [19] and linking with metal ions that then enhances plant tolerance to stress conditions. Proline improves metal-detoxification capacity of intracellular antioxidant enzymes. Under heavy metal stress, proline enhances antioxidant enzymatic activities, maintains cellular redox homeostasis, reconstructs Chl and regulates intracellular pH; thus proline acts as a metal chelator and protein stabilizer. Increase in proline depends on heavy metal concentration, toxicity threshold, plant organ and metal type [67]. Based on the obtained results, Cd toxicity did not change proline content probably due to high Cd toxicity. Cd, at lower dose, increased proline content due to increased ROS that led to adjusting cell osmotic pressure, preserving cell structures and providing energy and nitrogen required for cells [5, 57]. Hidangmayum et al. [50] stated that CTS might have impacts on proline production. Azimi et al. [5] also reported increase in proline content by CTS-Se NPs under non- and Cd- stress conditions. In addition, proline content was positively affected by CTS-Se NPs application under non- and salt- stress conditions [19]. Put acts in osmolyte accumulation, stabilizing cellular structures, triggering the antioxidant system and upregulating stress-related genes [20, 21] then, Put enhances proline production. Put and Put-CQD NPs enhanced proline under non- and salt- stress conditions [23]. PAs treatments (e.g., Put) caused enhancement in proline content in plants under salt-stress condition [65] due to its roles as nitrogen and energy supplier, ROS neutralizer and NADP+/NADPH redox state modulator that ultimately contributes to the preserving intercellular macromolecules and osmotic pressure [68]. Nahar et al. [53] and Shah et al. [59] reported increase in proline after Put treatment on plants under Cd-stress condition. Proline enhancement by CTS-Put NPs application could explain the reduced H_2_O_2_, MDA and EL under Cd-stress condition. CTS-Put NPs could contain CTS and Put impacts even stronger (like their role in proline biosynthesis or preventing its degradation possibly by affecting their genes or enzymes) due to using CTS as a carrier and functionalized nano-form and nano-size.

### Total anthocyanins and total phenolic compounds

**Fig. 7:**
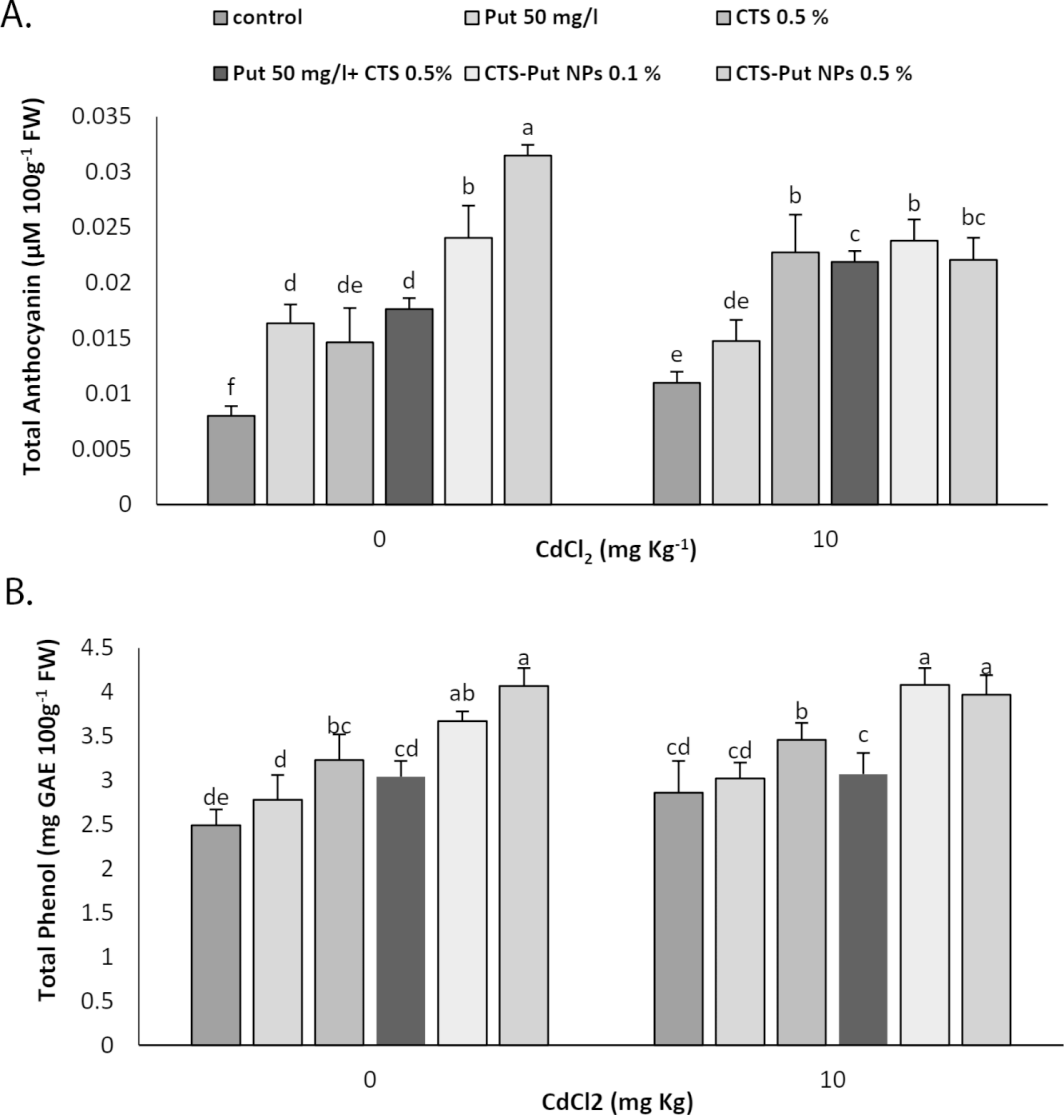
Effect of putrescine (Put; 50 mg L^− 1^), chitosan (CTS; 0.5%), “Put 50 mg L^− 1^ + CTS 0.5%” and “chitosan-putrescine nanoparticles” (CTS-Put NPs; 0.1 and 0.5%) treatments on total anthocyanins (**A**) and total phenolic compounds (**B**) of grapevines (*Vitis vinifera* L. cv. Sultana) under cadmium (Cd)-stress conditions (0 and 10 mg kg^− 1^). Same letters are not significantly different at *p* < 0.05

Cd stress increased total anthocyanins and total phenolics (Fig. 7). Regarding total anthocyanins, all treatments positively affected the content under non- and Cd- stress conditions except Put treatment under Cd-stress condition. The highest content of total anthocyanins were achieved by 0.5% CTS-Put NPs (Fig. 7A). Concerning total phenolics, CTS and both CTS-Put NPs treatments enhanced phenolics and the highest content was achieved by the NPs application under non- and Cd- stress conditions (Fig. 7B).

Phenolic compounds are non-enzymatic antioxidants capable of ROS and free radical detoxification and accordingly their content enhance under stress conditions to protect plant cells against stress adverse impacts [69]. Anthocyanins are derivatives of phenolic compounds with a similar ability to neutralize ROS and counteract their damaging effects by converting them into water molecules with their antioxidant roles [45]. Consequently, phenolic compounds and anthocyanins help plants to cope with stress conditions via quenching ROS and H_2_O_2_ [19] and their content increase under stress conditions [15]. Biosynthesis of phenolic compounds, flavonoids and anthocyanins could help tolerate or neutralize metal toxicity [70]. Cd primarily increased anthocyanin content to neutralize ROS [57]. Azimi et al. [5] reported increase in phenolic content under Cd-stress conditions. CTS enhanced anthocyanins and phenolics in nano-form [16]. CTS and CTS-Se NPs significantly enhanced phenolic content under Cd-stress conditions [5]. CTS-Se NPs enhanced anthocyanin, phenolic content and flavonoids under salinity condition [19]. CTS and CTS-Phe NPs application increased total anthocyanins, flavonoids and phenolic compounds and activity of phenylalanine ammonia-lyase (PAL), one of main enzymes responsible for their biosynthesis and accumulation [15]. CTS has elicitor-like activity particularly on the content of phenolic compounds, anthocyanins and antioxidant capacity via enhanced PAL activity [15, 16]. Put and Put-CQD NPs significantly enhanced phenolic compounds under non-stress condition while only Put-CQD NPs were successful under salt-stress condition [23]. Put application enhanced non-enzymatic antioxidants in plants under Cd-stress condition [63, 71]. PAs including Put enhanced phenolic compounds, anthocyanins and flavonoids [23, 68, 72], probably via increase in their biosynthetic enzymes or by preventing their degradation. Put is capable to increase non-enzymatic compounds and compatible osmolytes and antioxidant enzymatic activities [23] that could explain its positive role individually or in the CTS-Put NPs form on phenolics and anthocyanins. Therefore, CTS and CTS-Put NP effects on total anthocyanins and phenolic compounds might be related to enhancement in PAL and other enzymes in their biosynthesis and ROS scavenging that in turn could lessen Cd-induced oxidative stress.

### Antioxidant enzymes activities (APX, SOD, CAT, GP)

**Fig. 8:**
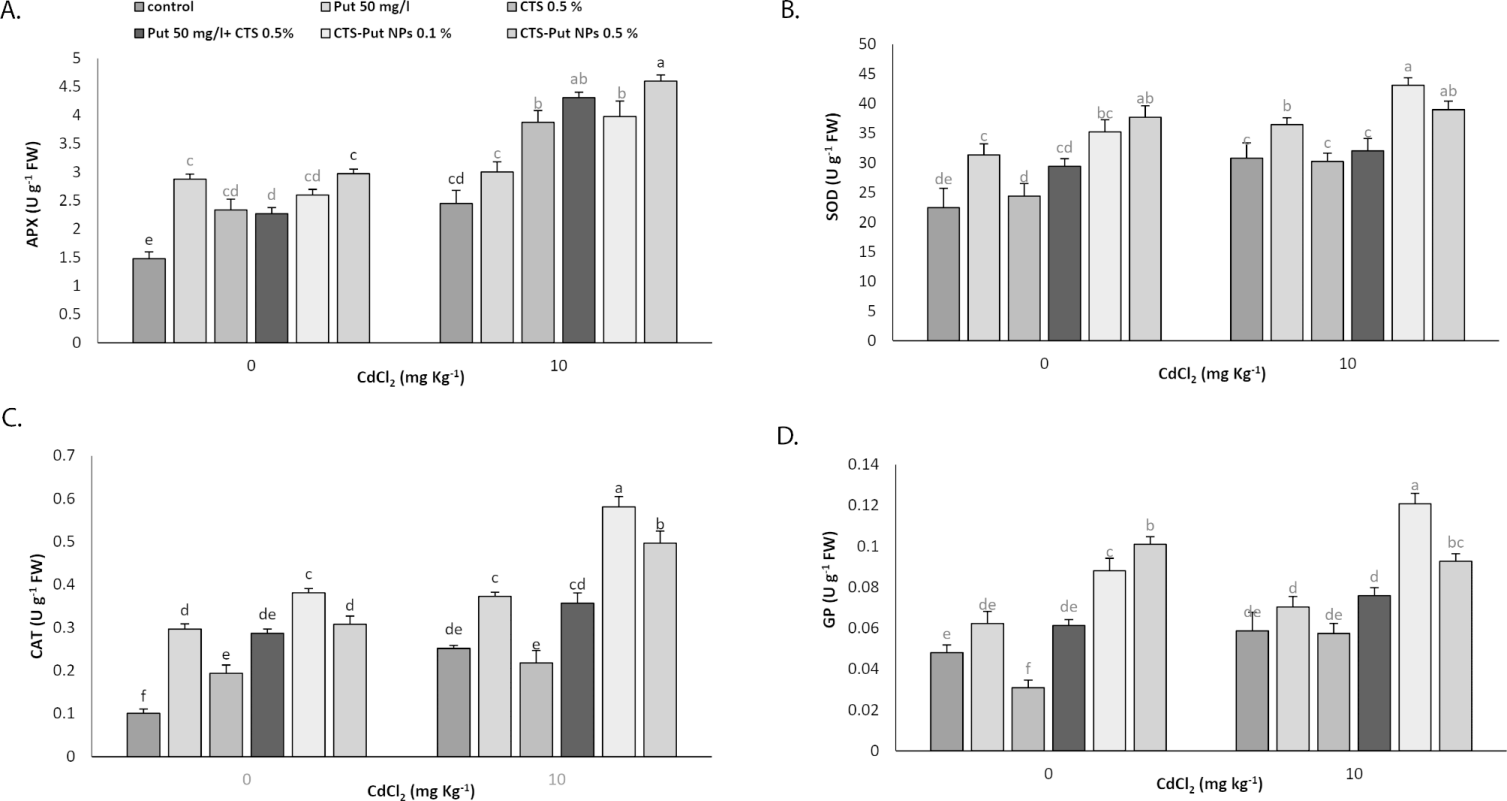
Effect of putrescine (Put; 50 mg L^− 1^), chitosan (CTS; 0.5%), “Put 50 mg L^− 1^ + CTS 0.5%” and “chitosan-putrescine nanoparticles” (CTS-Put NPs; 0.1 and 0.5%) treatments on ascorbate peroxidase (APX) (**A**), superoxide dismutase (SOD) (**B**), catalase (CAT) (**C**) and guaiacol peroxidase (GP) (**D**) of grapevines (*Vitis vinifera* L. cv. Sultana) under cadmium (Cd)-stress conditions (0 and 10 mg kg^− 1^). Same letters are not significantly different at *p* < 0.05

Cd stress significantly increased APX, SOD and CAT enzymes but GP did not significantly respond to the Cd stress (Fig. 8). All treatments enhanced APX enzyme activity under non- and Cd- stress conditions except Put under Cd-stress condition (Fig. 8A). SOD activity was enhanced by Put and both CTS-Put NPs treatments under non- and Cd- stress conditions (Fig. 8B). CAT activity was increased by application of all treatments under non-stress condition while Put and both CTS-Put NPs treatments had positive effects under Cd-stress condition (Fig. 8C). Under non- and Cd- stress conditions, CTS-Put NPs treatments (0.1 and 0.5%) enhanced GP activity (Fig. 8D). Mostly, 0.1% CTS-Put NPs acted as the best treatment under non- and Cd- stress conditions.

Cd stress damages plant cells over generating and accumulation ROS that induce oxidative stress and disturbs antioxidant defense system [73]. Antioxidant enzymes (e.g., CAT, SOD, APX, GP) protect cells from oxidant damage by buffering ROS damaging effects [5, 19]. In fact, antioxidant enzymes balance the ROS production and destruction [73]. The activity of SOD and POD decreased and increased under Cd-stress condition, respectively [74]. Azimi et al. [5] stated increase in APX, SOD, CAT and GP under Cd-stress condition. SOD, CAT and APX activities were increased under Cd-stress condition [74], as reported in the current study through probable greater activity of glutathione-ascorbate cycle [75]. CTS and CTS-Se NPs enhanced antioxidant enzymes activities under non- and Cd- stress conditions [5].

CTS-Se NPs [19] and Put and Put-CQDs NPs [23] application protected the plants against oxidative stress by enhancing CAT, POD, APX and SOD activities under non- and salt- stress conditions.

Put increases antioxidant enzymatic activities representing its role as a signaling molecule and stress-protective compound [72]. PAs could scavenge free radicals of cells as a direct free radical scavenger; improve cell survivability [76] and induce the expression of genes encoding antioxidant enzymes [26, 28, 29]. PAs bind to antioxidant enzyme molecules. Put positive effect on antioxidant enzymes was previously reported, contributing to neutralization of ROS [21] and stabilization of membrane structure [20]. Nahar et al. [53] reported enhanced APX, SOD and CAT enzymatic activities after Put application under Cd-stress condition. Similar results by Put application under Cd stress were reported by Taie et al. [70] for SOD and CAT. Put application enhanced and decreased CAT and SOD in plants under Cd-stress condition, respectively [71]. Put enhanced APX, CAT and SOD enzymes of the plants suffering from Cd stress [58]. CTS application activated antioxidant enzymes resulting in reduced ROS under stress condition [1, 51]. CTS could cause enhancement in the amount of plant polyamines that in turn could cause enhancement in Put function in activating antioxidant enzymes and antioxidant activity. Taking into account, CTS-Put NPs encouraging effects on the antioxidant enzymes could be explained with improved effectiveness and prolonged release of Put in the NPs form that at last removed Cd-induced oxidative stress. Nevertheless, more investigations are needed to clarify the real action mechanism.

## Materials and methods

### Location, plant materials and treatments

The study was accomplished in the research greenhouse of the Faculty of Agriculture, University of Tabriz (Tabriz, Iran), as a factorial experiment using a randomized design with four biological replications. The research farm soil was initially transferred to the greenhouse, sieved and then contaminated with cadmium chloride (CdCl_2_, Merck) as cadmium (Cd) source at different concentrations (0 and 10 mg kg^− 1^). Grapevines (*Vitis vinifera* L.) cv. Sultana (three-year-old cutting) were transferred to the polluted research farms soil and sand (3:1 ratio) (each pot (7-kg). The pots were irrigated every three days with the tap water until the harvest. Putrescine (Put; 50 mg L^− 1^), chitosan (CTS; 0.5%), “Put 50 mg L^− 1^ + CTS 0.5%” and “chitosan-putrescine nanoparticles” (CTS-Put NPs; 0.1 and 0.5%) were applied to the eight-leaf staged grapevine plants. The plants were sprayed four times with five days intervals. All measurements were performed four weeks after the application of the last treatments using fully expanded leaves. All measurements were performed in triplicate.

### Synthesis and characterization of chitosan-putrescine nanoparticles (CTS-Put NPs)

The synthesis of Put-CTS NPs was carried out according to our previous work, which was about the encapsulation of active metabolites using CTS [77]. Briefly, 1 g of low molecular weight CTS (100 kD, DD = 80%, Dr. Mahdavinia Co., Maragheh, Iran) was dissolved into 1000 mL of 0.1% wt of acetic acid solution to reach 0.1% wt of CTS solution. Then, 1 g of Put was added to homogeneous CTS solution and then the solution was stirred till complete dissolution of Put (~ 30 min). Afterwards, the Put-loaded CTS solution was treated with tripolyphosphate (TPP) solution (0.4 g dissolved in 20 mL distilled water) to obtain Put-CTS NPs. After TPP addition, Put-loaded CTS solution was sonicated to reach a homogeneous dispersion (the operating frequency used in sonication was 50 kHz).

### Photosynthetic pigments (chlorophyll a, b and carotenoids) and chlorophyll fluorescence parameters (^Fv^/_Fm_, Y (NO) and Y (II))

Fresh leaf samples (0.5 g) were extracted using acetone (3% v/v) and then followed by centrifuging (10,000 rpm, 10 min). The absorbance of supernatants were recorded by UV-Vis spectrophotometry (UV-1800 Shimadzu, Japan) and converted to the exact amounts of chlorophyll (chl) *a*, chl *b* and carotenoids [78]. Chlorophyll fluorescence parameters including ^*Fv*^*/*_*Fm*_, Y (NO) and Y (II) were estimated by a dual-pam-100 chlorophyll fluorometer (Heinz Walz, Effeltrich, Germany) after plant adaption in the dark [79].

### Leaf and root cd contents

The Cd content of leaf and root samples was determined according to the method of Azimi et al. [5] by atomic absorption spectrometer (Model CTA 3000, ChemTech, UK). Briefly, following the rinsing the samples with double-distilled water; the samples were dried in oven (65 °C, 48 h) and powdered. One gram of the samples was extracted with 10 ml of HNO_3_/HClO_4_ at 100 °C and the solution was then kept in a furnace (550 °C, 5 h) to obtain their ash. The obtained ashes were cooled and then dissolved with 10 ml HCL (2 N) and were filtered using Whatman filter paper. The final volume was completed to 50 mL with addition of double-distilled water.

### Malondialdehyde (MDA) and hydrogen peroxide (H_2_O_2_) content

MDA content quantified by addition of the equal amounts of thiobarbituric acid (0.5% w/v) in trichloroacetic acid (TCA) (20%) to the extracts of leaf samples in acetic acid (96 °C, 30 min). The solution was subsequently followed by an incubation period at 0 °C (5 min). Afterwards, the absorbance of the samples was recorded at 532 and 600 nm by the spectrophotometer. Finally, the content of was quantified [80]. For determination content of H_2_O_2_ in leaf samples, method of Sinha et al. [81] was employed. Briefly, leaf samples were extracted with trichloroacetic acid (0.1% w/v) at 0 °C and were followed by centrifugation. The supernatants were mixed with potassium phosphate buffer (pH 6.8, 10 mM) and potassium iodide (1 M). The absorbance of the samples was recorded at 390 nm by the spectrophotometer and the content was quantified using a standard curve obtained with different H_2_O_2_ concentrations.

### Electrolyte leakage (EL) and proline assay

The fresh leaf samples discs (0.5 cm) were washed by deionized water three times, placed in room temperate (24 h) and the initial electrical conductivity (EC_1_) was noted by a conductivity meter (Hanna, HI98192). Subsequently, they were placed in a water bath (95 °C, 20 min); cooled down (25 °C) and then their final electrical conductivity (EC_2_) was documented. EL was considered through EC1 and EC2 [82]. The electrolyte leakage rate was calculated according to the formula as follows: EL (%) = (EC_1_/EC_2_) x100.

After homogenizing leaf samples (0.5 g) with aqueous sulfosalicylic acid (10 mL, 3%) and centrifuging (1000 rpm, 4 °C), the supernatants were mixed with ninhydrin acid and glacial acetic acid (1:1:1); incubated (100 °C, 1 h) and then placed in an ice bath. Finally, after addition of toluene (4 mL) to the mixture, the spectrophotometrically noted absorbance (520 nm) was converted to proline contents using previously-made L-proline standard curve [82].

### Total anthocyanins and total phenolic compounds

For quantification of total anthocyanins, leaf samples were extracted with HCl–methanol (1:99) and then incubated at dark for 24 h. Following the incubation period, the samples were centrifuged. Finally, the absorbance of the samples was recorded at 550 nm [83].

Total phenolic content was quantified according to the method of Xu et al. [83]. In brief, leaf samples (0.1 g) were extracted using ethanol (5 mL, 95%) at dark for 24 h. The extracts were centrifuged and then the obtained supernatants (1 mL), ethanol (1 mL, 95%) and distilled water (3 mL) were mixed. Folin-Ciocalteu solution (0.5 mL, 50%) and sodium bicarbonate (1 mL, 5%) were added to the mixture, which were then incubated at dark for 1 h. The absorbance of the samples was recorded at 725 nm and the content was quantified with standard curve of Gallic acid.

### Assays of antioxidant enzymatic activity

Fresh leaf samples were first digested with potassium phosphate buffer (pH 6.8, 100 mM) containing polyvinylpyrrolidone (1%) and ethylenediaminetetraacetic acid (4 mM). The extraction was centrifuged (6000 rpm, 20 min, 4 °C). The supernatants were subsequently used to estimate the activity of ascorbate peroxidase (APX), catalase (CAT), superoxide dismutase (SOD) and guaiacol peroxidase (GP) enzymes [4, 23].

### Statistical analysis

Data were analyzed using one way variance analysis (SAS Institute Inc., ver. 9.1, Cary, NC, USA). The means were compared with Duncan’s multiple range test at *p* ≤ 0.05.

## Conclusion

Based on the former reports relating the affirmative effects of Put and CTS on cellular response of crops, a novel nano-conjugate (CTS-Put NP) was successfully synthesized and assayed for its potential uses in alleviating Cd-induced damage to grapevines. Accordingly, CTS-Put NP application significantly enhanced Chl *a*, *b*, carotenoids, ^*Fv*^*/*_*Fm*_, Y (II), proline, total phenolics, total anthocyanins and the activity of antioxidant enzymes and reduced Y (NO), leaf and root Cd content, EL, MDA and H_2_O_2_ under Cd-stress condition. Consequently, CTS-Put NPs, principally at lower dose (0.1%), could be introduced as an innovative ‘green’ approach with stress protecting properties in plant production, focusing on the alleviation of climate change-related abiotic stress conditions.

## Data Availability

The datasets used and/or analyzed during the current study are available from the corresponding author on reasonable request.
